# Testing a dual-process-inspired questionnaire-primary residual framework for personality prediction: an exploratory study with static line drawings

**DOI:** 10.3389/fpsyg.2026.1873771

**Published:** 2026-10-08

**Authors:** Lifen Weng, Yiyang Shi

**Affiliations:** 1 School of Design Art, Xiamen University of Technology, Xiamen, Fujian, China; 2 School of Computer and Information Engineering, Xiamen University of Technology, Xiamen, Fujian, China

**Keywords:** 16PF, dual-process theory, incremental validity, multimodal learning, static line drawings, personality prediction, questionnaire reduction

## Abstract

Comprehensive self-report personality inventories can impose substantial response burden, motivating the development of more efficient assessment strategies and the exploration of complementary information sources. Guided by Dual-Process Theory, this exploratory study examined a questionnaire-primary residual fusion framework in which a reduced questionnaire response vector served as the main predictive source, while static abstract line drawings were evaluated as a bounded auxiliary source of information. A total of 100 participants completed the 187-item Sixteen Personality Factor Questionnaire (16PF) and provided unprompted abstract line drawings. To account for the small sample size and reduce optimistic bias, we used participant-level repeated cross-validation, fold-local questionnaire item selection, standardized image preprocessing, cross-fitted residual learning, and nested selection of the visual correction scale. The reduced questionnaire provided a useful but imperfect held-out approximation of the full-form 16PF reference scores, with factor-level Pearson correlations ranging from 0.34 to 0.78. In contrast, the static drawing modality did not provide stable incremental predictive value. Nested validation selected a zero visual correction in 21 of 25 outer folds, and the fixed scale originally proposed for visual correction (0.05) produced a slightly higher mean absolute error than the questionnaire-only condition (1.2120 vs. 1.2100). An exploratory analysis using teacher-provided observer ratings likewise showed no advantage for visual fusion over questionnaire-only prediction. These findings indicate that Dual-Process Theory can motivate a testable asymmetric framework for integrating explicit and non-verbal information, but the present data do not support static final line drawings as a reliable source of incremental personality information. Future research should examine richer process-level drawing signals, including stroke timing, pauses, pressure, and trajectory dynamics, in larger independent samples.

## 1 Introduction

Personality assessment provides a structured means of describing relatively stable individual differences and is widely used in psychological research and educational settings. Comprehensive self-report inventories, including the 187-item Sixteen Personality Factor Questionnaire (16PF), can provide rich trait information, but their length can increase administration time and response burden. This has motivated interest in reduced assessment strategies. However, shortening a questionnaire is not merely a matter of retaining highly predictive items: a reduced item set may change construct coverage, factor stability, and measurement precision, so it requires independent validation rather than being assumed to preserve the psychometric properties of the original instrument (Cattell et al., 1970; Smith et al., 2000).

Self-report is also only one source of information about personality. Responses depend on what individuals are able and willing to report and can be affected by response style, self-presentation, fatigue, and other systematic sources of measurement variance (Paulhus, 1991). Indirect, behavioral, and observer-based information may therefore be considered as possible complements to self-report (Funder, 1995). Such information is not, however, inherently more accurate merely because it is less explicit. Any auxiliary modality requires its own evidence of reliability, construct validity, and incremental predictive value beyond direct self-report (Moeller et al., 2021). Evidence from implicit association tests further suggests that correspondence between explicit and indirect measures should be tested rather than presumed (Hasan et al., 2023).

This distinction is especially important for drawing-based psychological inference. Drawings have a long history as non-verbal or projective sources of psychological information, and spatial and morphological properties such as density, continuity, fragmentation, and coverage can be quantified computationally. At the same time, the validity of inferring stable personality characteristics from drawings remains contested; evidence for human figure drawings and the incremental validity of many projective indexes has been limited (Lilienfeld et al., 2000). Computational feature extraction does not itself establish psychometric validity: deep neural networks can learn visual features without validating a drawing-based inference for the intended psychological outcome (Lin et al., 2022). We therefore treat static abstract line drawings here as an exploratory non-verbal modality rather than as an established implicit personality measure.

Static abstract line drawings were selected because they can be collected with little specialized equipment while retaining basic spatial and morphological information. They omit process-level signals such as stroke timing, pauses, pressure, drawing order, and trajectory dynamics, which may be available in handwriting-dynamics, response-latency, voice, or eye-tracking paradigms. They are therefore treated as a deliberately simple exploratory candidate modality, not as an optimal or validated implicit measure.

Computational personality research has increasingly considered information from text, audio, visual behavior, and other modalities (Zhao et al., 2022). Multimodal machine learning provides tools for representing and combining such heterogeneous data, while also emphasizing challenges of modality interaction and transfer (Baltrušaitis et al., 2019). Modalities can differ substantially in predictive strength, reliability, and sample efficiency. Consequently, a weak or noisy modality may worsen generalization rather than contribute useful complementary information, particularly in small psychological datasets where high-capacity visual models may fit sample-specific variation.

Guided by Dual-Process Theory, we therefore examine a deliberately asymmetric integration hypothesis. Dual-process accounts distinguish more reflective and controlled processing from more automatic and less deliberative processing (Evans and Stanovich, 2013). Questionnaire responses rely strongly on explicit reflection and verbalized self-description, whereas spontaneous non-verbal behavior may involve partially different processes. This distinction does not establish the validity of a particular non-verbal measure. Instead, it motivates a conservative architectural prior: the questionnaire remains the principal predictive anchor, and a candidate visual modality is permitted only a bounded auxiliary correction if it contains reproducible incremental information.

In computational terms, the final Sten-scale prediction is expressed as *ŷ*_fused, Sten = clip(*q*_Sten + *αr̂*_Sten, 1, 10), where *q*_Sten is the questionnaire prediction, *r̂*_Sten is the Sten-scale visual residual prediction, and α limits the visual contribution. The theoretical role of this formulation is to specify how information sources should be combined, rather than to assume in advance that the visual residual represents subconscious or more authentic personality information.

The present exploratory study evaluates this framework in 100 participants who completed the full 187-item 16PF and provided unprompted abstract line drawings. A data-driven reduced questionnaire response vector was used as the primary predictive input, while standardized static drawings were evaluated as a candidate auxiliary modality. We asked three open research questions: RQ1: To what extent can a reduced questionnaire response vector approximate held-out full-form 16PF reference scores? RQ2: Do static abstract line drawings provide reproducible incremental predictive information beyond the questionnaire-only model? RQ3: If visual correction is permitted, what correction magnitude is supported under leakage-controlled, participant-level validation?

## 2 Materials and methods

### 2.1 Participants, data collection, and ethics

The analytic sample comprised 100 participants drawn from high-school and undergraduate educational settings. Questionnaires were collected through those settings; part of the material originated from paper questionnaires that were subsequently digitized, and some responses were collected electronically. Incomplete questionnaires were excluded during the original data preparation. Detailed participant-level demographic variables, including age and sex, were unavailable for the present analyses and are therefore not reported. The retained records did not include an exact participant-level recruitment mode, the original exclusion threshold, or a transcription quality-control record for the paper forms.

The study was approved by Xiamen University of Technology (project number 2026Y3007; protocol or registration number (2026) Registration No. (7); approval date 23 February 2026). Written informed consent was obtained before data collection.

### 2.2 Full-form 16PF reference scores

The Chinese-language questionnaire contains 187 items with three response options and item-specific scoring keys. Formal scoring and model-input encoding were treated as separate operations. For formal scoring, the retained scoring keys were applied to the designated items for the 16 primary dimensions, and the resulting raw scores were converted to 1–10 Sten scores using the supplied conversion table. These full-form Sten values are called reference scores throughout this article. Items 1, 2, and 187 are not included in the formal scoring of the 16 primary factors.

The primary factors were A (Warmth), B (Reasoning), C (Emotional Stability), E (Dominance), F (Liveliness), G (Rule-Consciousness), H (Social Boldness), I (Sensitivity), L (Vigilance), M (Abstractedness), N (Privateness), O (Apprehension), Q1 (Openness to Change), Q2 (Self-Reliance), Q3 (Perfectionism), and Q4 (Tension). Here, Q1–Q4 denote factor labels, whereas item 1 and item 2 denote questionnaire items. The retained study records include item wording, scoring keys, and the Sten conversion table. The exact Chinese adaptation/edition and normative provenance could not be independently verified from the retained study records.

### 2.3 Data-driven reduced questionnaire representation

For prediction, each response option was encoded as A = 2, B = 1, and C = 0, yielding an initial 187-dimensional numeric questionnaire-response vector. This vector was not natural-language text. Within every outer-training partition, a Ridge regression (*α* = 10) was fit separately for each of the 16 reference-score dimensions. The two items with the largest absolute regression coefficients were retained for each factor, and their union formed the fold-local reduced questionnaire response vector. The resulting input dimension was 31–32 items, depending on the outer fold. Selected item sets could therefore differ across folds. The Ridge penalty was fixed at 10 across all fold-local Ridge fits and was not selected using outer-test performance.

Each selected response feature was standardized using the mean and standard deviation estimated only from the relevant training partition. No outer-test participant contributed to selection or feature scaling.

### 2.4 Static line-drawing acquisition and preprocessing

Participants provided spontaneous abstract line drawings. The collection instructions asked participants to avoid concrete objects and written text. The original images varied in size, framing, illumination, paper or background color, and pen color. These acquisition differences motivated a single label-blind preprocessing procedure; drawings were not manually edited or excluded on the basis of their appearance or model results.

For each image, EXIF orientation was applied, the image was converted to RGB, and its longest side was limited to 512 pixels. Illumination and color cast were normalized by dividing each RGB channel by a Gaussian-blurred background estimate (radius = 35), with denominator floor 8, and multiplying by 255. The normalized image was converted to grayscale using 0.2126R + 0.7152G + 0.0722B. A line mask was then obtained from local darkness within a radius-9 neighborhood: a pixel was retained when local mean minus grayscale intensity exceeded max(3, 0.35 times the local mean absolute deviation). The mask was dilated by one pixel. Corrected continuous grayscale intensity was retained within the mask; all other pixels were set to white (255). The image was resized with aspect ratio preserved and placed on a white 128 × 128 canvas using Lanczos resampling.

The resulting clean-gray standardized image was the main visual input. Binary and skeleton versions were generated only for preprocessing quality control and ablation purposes. Quality-control summaries included background mean and standard deviation, foreground ratio, edge energy, connected components, skeleton length, and line bounding-box coverage. Possible text or annotation contamination was flagged rather than removed. EXIF or GPS metadata were not model inputs; a separate metadata-stripping procedure was not part of the analysis pipeline.

### 2.5 Questionnaire-primary residual architecture

The model was specified as a questionnaire-primary, sequential residual architecture. Let *y_*Sten denote a full-form reference score on the 1–10 Sten scale. The visual term is a questionnaire-prediction residual correction, not a latent psychological construct. The following equations state the implemented unit conversions and fusion directly.


$$y_{\mathrm{norm}}=\frac{y_{\text{Sten}}-1}{9}$$

$$q_{\mathrm{norm}}=f\_Q(x),\;\;\;L\_Q=\mathrm{MSE}(q_{\mathrm{norm}},y_{\mathrm{norm}})$$

$$q_{\text{Sten}}=1+9q_{\mathrm{norm}}$$

$$r_{\text{Sten}}=y_{\text{Sten}}-q\_{\text{Sten}}^{\mathrm{OOF}}$$

v=tanh(f_V(I)),v∈[−1,1]

r^Sten=9v

LV=MSE(r^Sten,rSten)=MSE(9v,rSten)

y^fused,Sten=clip(qSten+αr^Sten,1,10)

An exactly equivalent normalized-space expression is *q*_fused,norm = clip(*q*_norm + *αv*, 0, 1), followed by *ŷ*_fused, Sten = 1 + 9*q*_fused,norm. Thus, the raw tanh output *v* is not a Sten residual; 9*v* is the Sten-scale predicted residual. Both the cross-fitted residual target and the visual prediction used for loss evaluation and fusion are in Sten units. The released implementation follows these equations directly: the questionnaire prediction is converted back to the Sten scale before residual construction, the TinyCNN tanh output is multiplied by 9 before both residual-loss evaluation and fusion, and final fusion is clipped to the 1–10 Sten range.^1^

The Questionnaire Tower was a multilayer perceptron with k input features (*k* = 31 or 32), a 48-unit linear layer, ReLU activation, dropout (*p* = 0.20), and a 16-unit linear output layer followed by a sigmoid. It had 2,320 trainable parameters when *k* = 31 and 2,368 when *k* = 32. The TinyCNN used for the primary visual residual analysis accepted a 1 × 128 × 128 clean-gray image and contained 3 × 3 convolutions with 16, 32, and 48 channels; batch normalization and ReLU followed each convolution; the first two blocks included 2 × 2 max pooling. A 1 × 1 convolution produced 16 factor-specific maps, which were global-average-pooled and passed through tanh. The Questionnaire Tower contained 2,320 parameters for 31 input items and 2,368 parameters for 32 input items. The TinyCNN visual residual tower contained 19,648 trainable parameters. The complete questionnaire-plus-visual architecture therefore contained 21,968–22,016 total parameters, depending on whether 31 or 32 questionnaire items were selected in that fold.

### 2.6 Leakage-controlled repeated cross-validation

Participants were the splitting unit. We used five repeats of five-fold outer cross-validation, giving 25 fixed outer folds. Each fold trained on 80 participants and held out 20 participants, and each participant appeared once as an outer-test participant per repeat. The fixed outer-fold seeds were 1,101, 2,202, 3,303, 4,404, and 5,505. The same assignments were used across model conditions. All primary analyses used this repeated participant-level cross-validation design.

All questionnaire selection and scaling were fit inside the appropriate training partition. Image preprocessing used a fixed label-blind rule set; it did not use outcome labels. Outer-test participants were not used for item selection, scaler fitting, residual-target construction, model selection, or scale selection. Programmatic seeds set Python random, NumPy, PyTorch CPU, and PyTorch CUDA generators; fold-specific seed formulas were specified in the analysis scripts and configuration files.

### 2.7 Cross-fitted visual residual learning

For each outer fold, the reduced questionnaire model was cross-fitted within the outer-training participants using a shuffled five-fold inner split. In every inner fit, item selection, scaling, and Questionnaire Tower fitting used only the inner-training participants. Each outer-training participant therefore received a questionnaire prediction from a model that had not trained on that participant. The visual target was the out-of-fold questionnaire-prediction residual below. This residual is a prediction error of the questionnaire model, not a latent psychological construct.


$$r_{i,\text{Sten}}=y_{i,\text{Sten}}-q_{i,\text{Sten}}^{\mathrm{OOF}}$$

TinyCNN was trained on the outer-training clean-gray images to predict these cross-fitted residuals using mean squared error in Sten units. For outer testing, the questionnaire model was refit on the full outer-training partition, and the previously trained visual model supplied the bounded Sten-scale residual prediction. Fusion then used the predefined α and Sten-scale clipping to [1, 10]. A morphology Ridge control using the same cross-fitted residual targets and fold-local feature standardization is retained as a supplementary sensitivity analysis.


$$L_{V}=\frac{1}{N}\Sigma_{i}{\left\|{\overset{\wedge }{r}}_{i,\text{Sten}}-r_{i,\text{Sten}}\right\|}^{2}$$

$${\overset{\wedge }{r}}_{i,\text{Sten}}=9v_{i}$$

### 2.8 Training details, scale selection, and sensitivity analysis

Questionnaire models were optimized with Adam (learning rate 0.001, weight decay 0.0001, batch size 16) for 80 epochs using normalized-score mean squared error. TinyCNN was optimized with Adam (learning rate 0.0005, weight decay 0.0001, batch size 16) for 100 epochs. No data augmentation was applied. Models were trained for fixed numbers of epochs; early stopping was not used. Questionnaire and TinyCNN parameters used the framework’s default initialization.

The fixed correction scale was *α* = 0.05. Because the raw TinyCNN output v is bounded between −1 and 1 and the Sten-scale residual prediction is *r̂*_Sten = 9v, *α* = 0.05 corresponds to a maximum correction of ±0.45 Sten. The nested analysis used the predefined candidate grid 0, 0.005, 0.01, 0.025, 0.05, 0.075, and 0.10, where 0 is the no-visual-correction condition. Within each outer-training partition, a five-fold inner procedure selected the scale with the lowest fused validation MAE; exact ties were resolved in favour of the smaller scale. Outer-test data were used only after this choice. The fixed-scale curve was retained as a descriptive sensitivity analysis and was not used to retrospectively choose a scale.

The analyses used Python 3.10.19, PyTorch 2.0.0 + cu117, torchvision 0.15.0 + cu117, and CUDA on an NVIDIA GeForce RTX 3050 Ti Laptop GPU. The available environment information lists NumPy, pandas, scikit-learn, SciPy, Pillow, OpenCV, and Matplotlib; exact version numbers for those packages and the CUDA toolkit version were unavailable.

### 2.9 Teacher-provided external criterion

A secondary observer analysis used a teacher-provided external criterion for 15 participants. Each participant was rated by one familiar homeroom teacher, who nominated three salient dimensions from the same 16-factor set. The retained records do not identify class membership, teacher identity, the number of contributing teachers, blinding procedures, or the rating instrument, and they do not encode high-versus-low polarity. Because no participant had multiple independent raters, inter-rater reliability could not be estimated and teacher and class effects could not be separated. Accordingly, this analysis assessed dimension-level salience alignment only; it did not assess directional agreement.

For a predicted 16-factor Sten vector, salience was defined by the expression below, and the three largest values were selected with a stable factor-order tie rule. We calculated overlap count, any-hit, exact-match, and Jaccard similarity. Because both sets had size three, precision at 3 and recall at 3 equaled overlap/3. Chance comparisons used 20,000 Monte Carlo draws of three factors from the 16-factor set for each participant. Questionnaire-only, fixed-α fusion, and nested-fusion predictions were compared with paired permutation tests and Wilcoxon tests for overlap and Jaccard, and an exact McNemar test for any-hit when discordant pairs existed.


$$s_{\mathrm{ij}}=|y_{\mathrm{ij},\text{Sten}}-5.5|$$

### 2.10 Evaluation metrics and statistical analysis

The primary prediction metric was mean absolute error (MAE) in Sten units. Mean squared error was used for model optimization. Reduced-questionnaire correspondence was summarized with factor-wise Pearson *r*, Spearman rho, MAE, root mean squared error, and *R*-squared, with bootstrap confidence intervals for the correlations. A Bland–Altman-style plot was treated as an exploratory visualization because Sten scores are bounded and discrete. Correlation was interpreted as predictive correspondence rather than agreement or structural validity.

The participant remained the inferential unit for paired statistical comparisons, whereas MAE aggregation was reported using the specific prediction-level estimand stated for each analysis. Two non-interchangeable MAE estimands were used. For participant-averaged held-out MAE, five held-out predictions were first averaged for each participant-factor and absolute error was then computed. For held-out-appearance MAE, absolute error was computed separately for every repeat-specific held-out prediction before averaging. Primary paired analyses reported the mean and median difference, bootstrap 95% confidence interval, paired t test, Wilcoxon signed-rank test, and paired Cohen d. Bootstrap intervals used 20,000 participant-level resamples. Factor-specific analyses reported all 16 factors as secondary, exploratory results; no multiplicity correction was applied and they were not interpreted as independent confirmatory tests.

Generative-AI tools assisted code auditing, debugging, and workflow implementation. All final analytical code, statistical outputs, figures, references, and interpretations were reviewed and approved by the authors, who take full responsibility for the accuracy and integrity of the manuscript.

For Estimand A (participant-averaged held-out MAE), *N* = 100 and *q̄*_*ij* is the average of the five held-out predictions for participant i and factor j.


$${\bar{q}}_{\mathrm{ij}}=(1/5)\;\Sigma \_rq_{\mathrm{ijr}}$$

$${\mathrm{MAE}}_{\mathrm{PA}}=\frac{1}{N\times 16}\Sigma_{i}\Sigma_{j}|y_{\mathrm{ij}}-{\bar{q}}_{\mathrm{ij}}|$$

For Estimand B (held-out-appearance MAE), *e*_*ijr* is the absolute error for a repeat-specific held-out prediction. Because all outer folds contain equal numbers of participants, this equals the unweighted mean outer-fold held-out MAE.


$$e_{\mathrm{ijr}}=|y_{\mathrm{ij}}-q_{\mathrm{ijr}}|$$

$${\mathrm{MAE}}_{\mathrm{HA}}=(1/(N\times 16\times 5))\;\Sigma \_i\;\Sigma \_j\;\Sigma \_re_{\mathrm{ijr}}$$

Nested alpha selection was conducted only within outer-training data. For each outer fold, *O*_train was divided into five inner folds. Within each *I*_train, the Questionnaire Tower was cross-fitted again to construct *I*_train residual targets; fold-local item selection, scaling, and questionnaire fitting used *I*_train only; TinyCNN was trained on *I*_train images and those cross-fitted targets; and each candidate α was evaluated on *I*_val only. The α minimizing mean inner-validation MAE was selected, after which the residual targets and both towers were reconstructed using all *O*_train and *O*_test was evaluated once. No inner-validation participant contributed to the fitting of the TinyCNN used to generate that participant’s visual prediction. Outer-test participants were excluded from item selection, scaler fitting, residual-target construction, questionnaire fitting, TinyCNN fitting, and alpha selection.

The questionnaire-primary residual evaluation framework is shown in Figure 1.

**Figure 1:**
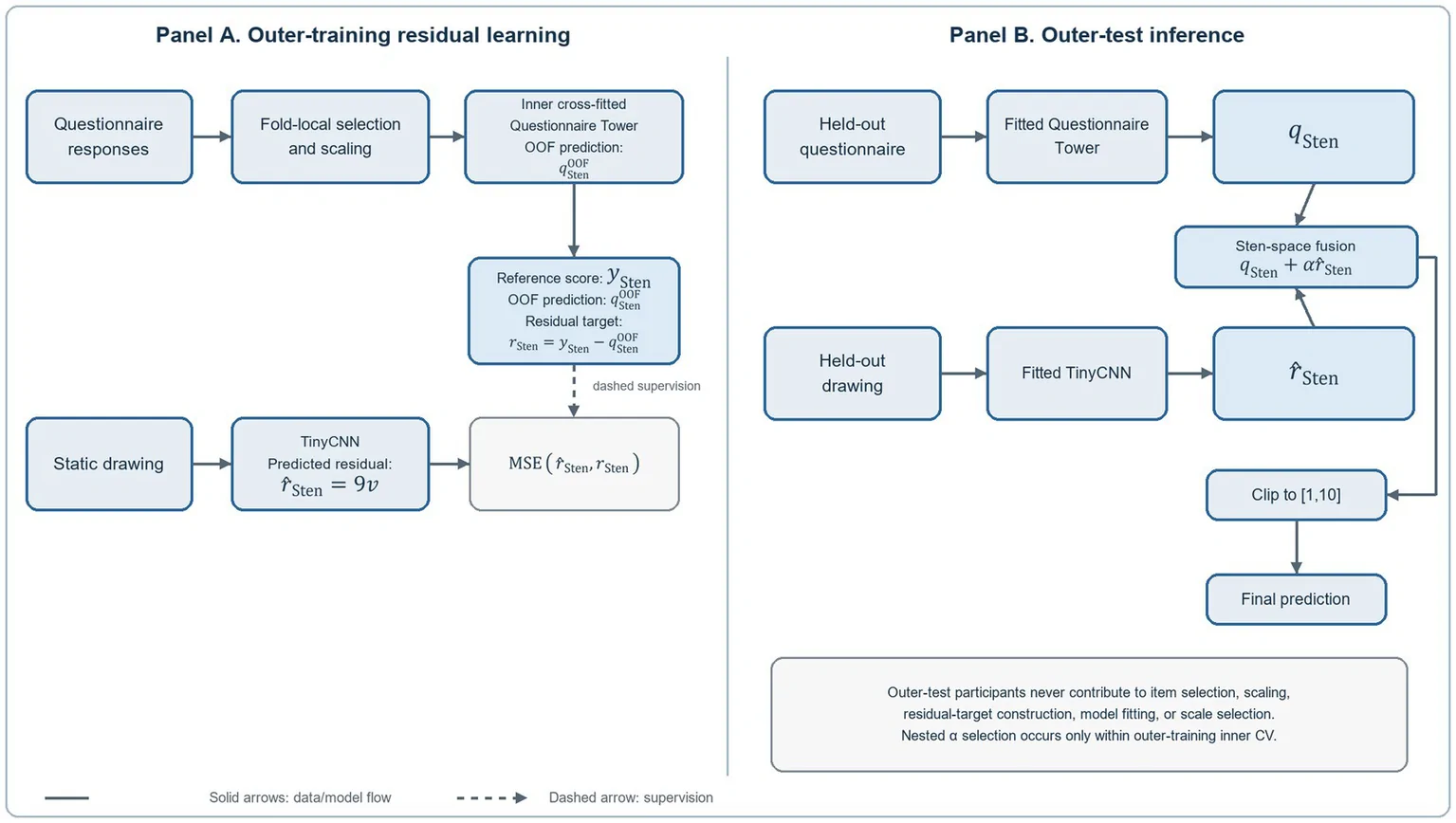
Questionnaire-primary residual evaluation framework. Panel **(A)** depicts cross-fitted residual learning within the outer-training data: fold-local questionnaire selection and scaling support inner cross-fitted Questionnaire Tower predictions, which form the residual target with the reference score. Static drawings enter TinyCNN, which predicts that residual; dashed arrows denote supervision through the MSE loss. Panel **(B)** depicts held-out inference. Nested α selection occurs only within outer-training inner cross-validation. Outer-test participants are excluded from selection, scaling, residual-target construction, model fitting, and scale selection.

## 3 Results

### 3.1 Reduced questionnaire correspondence

Across the 100 participants, questionnaire-only held-out predictions from the repeated cross-validation analysis showed useful but imperfect correspondence with the full-form 16PF reference scores. For each participant-factor, five held-out predictions were averaged before error was computed. The participant-averaged held-out MAE was 1.1121 Sten [median = 1.1130; bootstrap 95% CI (1.0576, 1.1654)]. Across pooled participant-by-factor cells, the RMSE was 1.3909 and the descriptive *R*^2^ was 0.4717; these pooled values are descriptive because factor cells within participants are not independent (see Figure 2).

**Figure 2:**
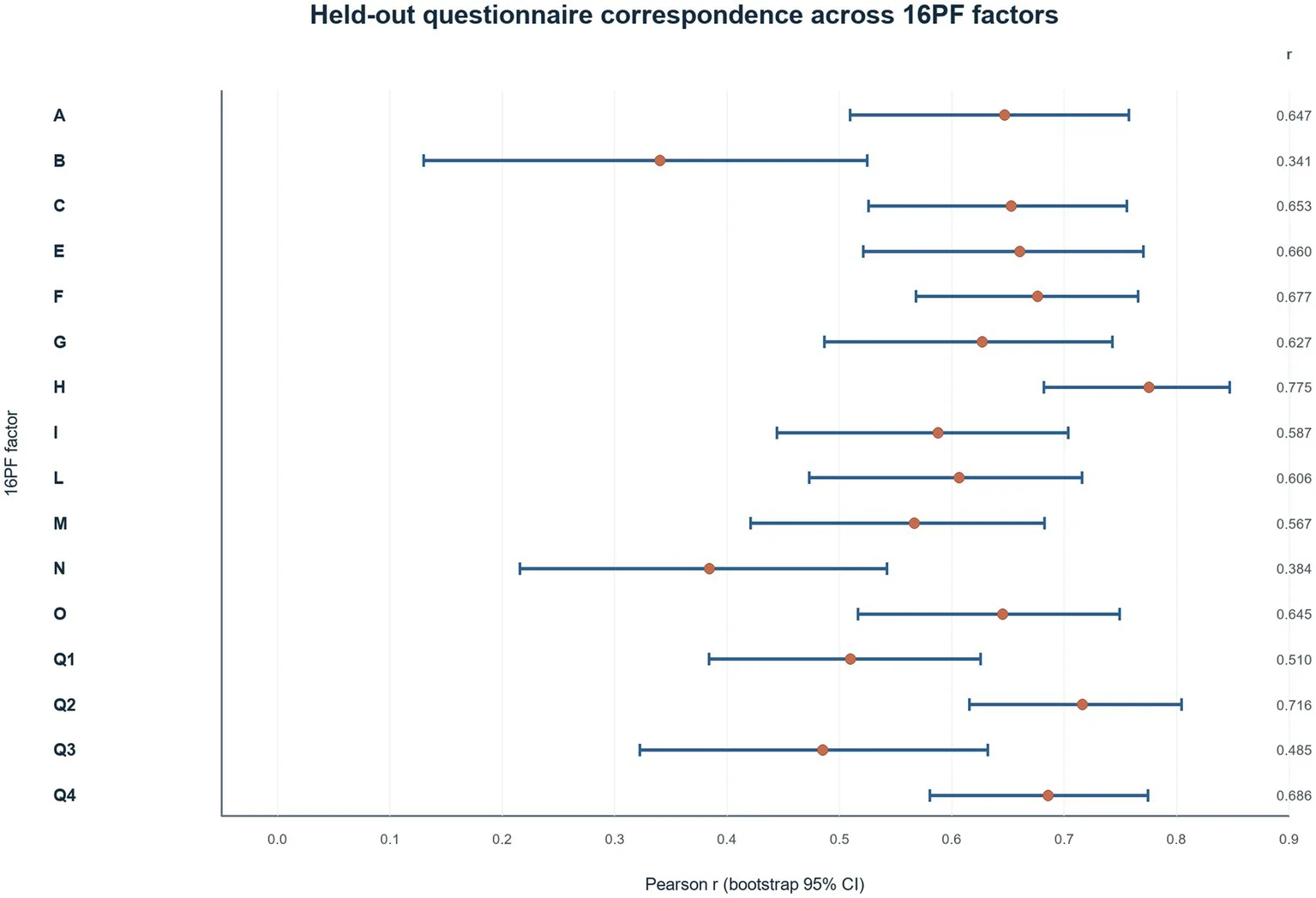
Held-out questionnaire correspondence across 16PF factors. Pearson correlations and bootstrap 95% confidence intervals are shown for the 16 full-form 16PF reference-score dimensions after averaging five held-out predictions per participant (*n* = 100). Correlation indicates predictive correspondence, not structural validity.

Factor-level Pearson correlations ranged from 0.341 to 0.775, indicating heterogeneous correspondence across the 16 factors. The highest correlation was for *H* [*r* = 0.775, 95% CI (0.682, 0.847)] and the lowest was for *B* [*r* = 0.341, 95% CI (0.130, 0.524)] (Table1).

**Table 1:** Held-out correspondence between reduced-questionnaire predictions and full-form 16PF reference scores.

Factor	Pearson *r* (95% CI)	Spearman *ρ*	MAE	RMSE	*R* ^2^
A	0.647 [0.509, 0.757]	0.645	1.006	1.280	0.400
B	0.341 [0.130, 0.524]	0.282	1.384	1.702	0.114
C	0.653 [0.526, 0.756]	0.631	0.939	1.168	0.420
E	0.660 [0.521, 0.770]	0.648	0.929	1.261	0.426
F	0.677 [0.568, 0.765]	0.640	1.136	1.415	0.434
G	0.627 [0.486, 0.743]	0.619	1.075	1.365	0.364
H	0.775 [0.682, 0.847]	0.756	1.041	1.257	0.583
I	0.587 [0.444, 0.703]	0.553	1.235	1.541	0.331
L	0.606 [0.473, 0.715]	0.545	1.174	1.398	0.352
M	0.567 [0.421, 0.682]	0.533	0.994	1.266	0.312
N	0.384 [0.215, 0.542]	0.368	1.183	1.427	0.145
O	0.645 [0.516, 0.749]	0.629	1.307	1.554	0.403
Q1	0.510 [0.383, 0.625]	0.519	1.333	1.612	0.255
Q2	0.716 [0.615, 0.804]	0.729	1.180	1.468	0.488
Q3	0.485 [0.322, 0.632]	0.461	0.957	1.228	0.233
Q4	0.686 [0.580, 0.774]	0.702	0.919	1.172	0.455

*n* = 100 participant summaries; five held-out questionnaire predictions were averaged per participant. Correlations indicate predictive correspondence, not structural validity.

The fold-local response sets contained 31–32 items; the selected response sets varied across folds (mean pairwise Jaccard = 0.414, range = 0.231–0.600). Item 187 was selected in 16 of 25 folds, whereas Items 1 and 2 were not selected in any fold.

### 3.2 Cross-fitted visual residual predictability

Direct residual prediction was evaluated using the held-out questionnaire and visual residual predictions after averaging the five held-out predictions per participant-factor. The residual was defined as the full-form reference Sten score minus the questionnaire prediction.

For the participant-averaged held-out residual analysis, the zero-residual predictor had MAE = 1.112 and MSE = 1.935; TinyCNN had MAE = 1.227 and MSE = 2.364. In the participant-level comparison (*n* = 100), TinyCNN-minus-zero residual MAE was +0.115 Sten [95% CI (0.084, 0.150); *p* < 0.001 by paired *t* test; *p* < 0.001 by Wilcoxon test; *d* = 0.68], where a positive difference indicates higher error. TinyCNN improved residual MAE for 22 participants and worsened it for 78 (Table 2).

**Table 2:** Held-out visual-residual prediction.

Predictor	MAE	MSE	Δ MAE vs. zero (95% CI)
Zero residual	1.112	1.935	—
TinyCNN	1.227	2.364	+0.115 [+0.084, +0.150]

Participant-level comparisons average five held-out predictions and then average across 16 factors (*n* = 100). Positive differences indicate higher error.

TinyCNN direction accuracy was 0.486 and balanced accuracy was 0.486 for the 1,382 residual cells with absolute magnitude at least 0.25 Sten. Pooled descriptive associations were near zero (Pearson *r* = −0.055; Spearman rho = −0.048); these summaries are descriptive because factor cells are nested within participants. A separately refitted morphology-based control and a residual-target diagnostic are reported in the Supplementary material.

### 3.3 Bounded fusion and scale selection

The fixed-scale sensitivity analysis showed that all non-zero scales increased MAE relative to the questionnaire-only model. In the predefined nested-CV fusion analysis, the mean held-out-appearance MAE was 1.2103 [95% CI (1.1547, 1.2641)], compared with 1.2100 [95% CI (1.1544, 1.2638)] for the questionnaire-only model. The participant remained the inferential unit for the paired comparison, which yielded a difference of +0.00022 [95% CI (0.00003, 0.00042); paired *t*-test *p* = 0.032; Wilcoxon signed-rank *p* = 0.033; Cohen’s *d* = 0.22]. Nested fusion tied the questionnaire-only model in the 21 outer folds for which *α* = 0 was selected and produced higher MAE in the four folds selecting a non-zero scale; it produced lower MAE in none of the 25 outer folds.

In the descriptive fixed-scale sensitivity analysis, held-out-appearance MAE was 1.21004 at *α* = 0, 1.21017 at 0.005, 1.21032 at 0.01, 1.21086 at 0.025, 1.21200 at 0.05, 1.21341 at 0.075, and 1.21516 at 0.10. All tested nonzero scales had higher held-out-appearance MAE than *α* = 0. Figure 3 displays the fixed-scale sensitivity and nested selection frequency.

**Figure 3:**
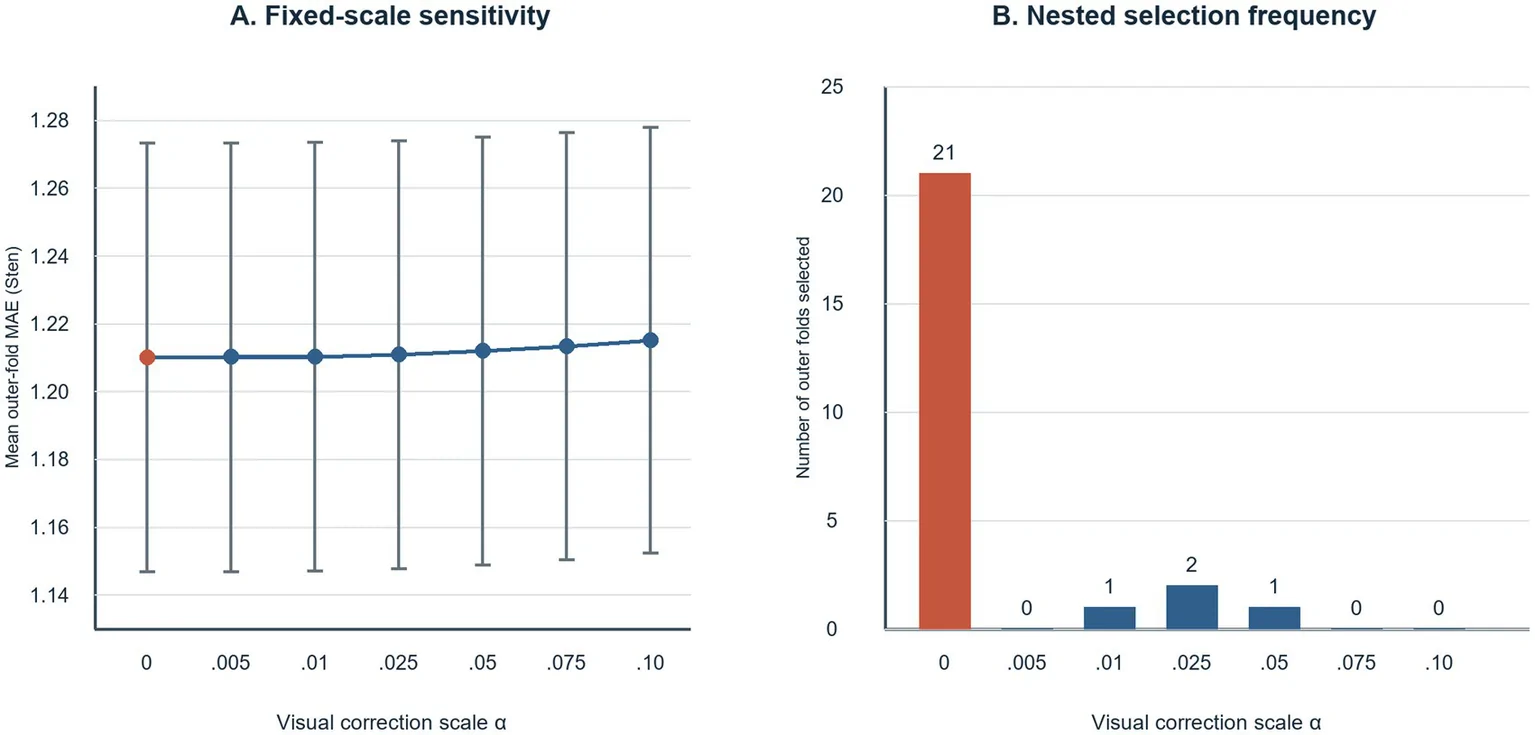
Fixed-scale held-out MAE and nested selection frequency. In Panel **(A)**, points show mean outer-fold MAE and error bars show the descriptive standard deviation across the 25 outer folds. Panel **(B)** shows nested inner-cross-validation selection frequency across 25 outer folds.

### 3.4 Teacher-provided external criterion

The secondary teacher-provided external observer analysis included 15 participants. All 15 teacher records were reliably mapped, each contained three trait nominations, no duplicate participant IDs were present, and held-out predictions were available for every mapped participant.

For the full-form self-report reference, mean overlap with the teacher-nominated salience set was 0.800 and mean Jaccard similarity was 0.180. The corresponding one-sided Monte Carlo chance-comparison *p* values were 0.109 for overlap and 0.076 for Jaccard. For the held-out questionnaire prediction, mean overlap was 0.467 and mean Jaccard similarity was 0.107; chance-comparison *p* values were 0.782 and 0.658, respectively.

At *α* = 0, the fusion equation reduces exactly to the questionnaire-only prediction. Accordingly, under the same aggregation estimand, the *α* = 0 and questionnaire-only predictions and MAEs are numerically identical. The apparent difference between the reported 1.1121 and 1.2100 values reflects different aggregation orders, not different questionnaire predictions.

For the 15 observer-rated participants, fixed *α* = 0.05 fusion and nested fusion produced the same Top-3 salience sets as questionnaire-only prediction. Accordingly, the paired differences in overlap count, Jaccard similarity, and any-hit were all 0, and visual fusion produced no incremental observer alignment.

## 4 Discussion

### 4.1 Summary of the main findings

The reduced questionnaire provided a useful but imperfect held-out approximation of the full-form 16PF reference scores. Across factors, correlations ranged from 0.34 to 0.78, and the participant-averaged held-out MAE was approximately 1.11 Sten units. The selected response sets also varied across folds (mean pairwise Jaccard = 0.414), indicating that the retained items should not be interpreted as a stable short-form solution. These findings support only a limited, sample- and procedure-specific form of questionnaire reduction.

Static line drawings did not improve prediction beyond this questionnaire baseline. For the participant-averaged held-out residual-prediction analysis, TinyCNN residual MAE was approximately 1.23 Sten units, compared with approximately 1.11 for the zero-residual predictor (ΔMAE = +0.115). Direction accuracy was approximately at chance level (0.486). In the nested fusion analysis, *α* = 0 was selected in 21 of 25 outer folds; the four non-zero selections worsened held-out-appearance MAE, and no fold improved it. All tested non-zero correction scales yielded higher held-out-appearance MAE than the no-correction condition.

The small teacher-rated exploratory analysis did not reverse this pattern. Questionnaire-only and fusion predictions identified the same three most salient factors, so it supplied no evidence that the visual branch added observer-relevant information. Teacher ratings are not treated here as a criterion truth measure: each participant had one familiar teacher rating, rather than multiple independent raters.

### 4.2 Reduced questionnaire prediction: useful correspondence but not a validated short form

The selected response subset should not be described as a validated 16PF short form. Its performance concerns correspondence with the retained full-form scoring procedure in this dataset, not equivalence to an established short form or preservation of the construct coverage intended by the full instrument (Cattell et al., 1970; Smith et al., 2000). Although Item 187 was repeatedly selected, repeated selection is not evidence that the item measures response quality, a separate psychological construct, or any special latent mechanism.

A defensible short-form claim would require preregistered item content review, replication in independent samples, reliability and factor-structure evidence, tests of measurement invariance, and criterion-related validation against appropriate external outcomes. Those requirements were not met in the present study. The reduced questionnaire is therefore best understood as an exploratory predictive subset rather than a replacement assessment.

### 4.3 No stable incremental value from static line drawings

Several analyses converged on the same negative conclusion. The visual residual model did not outperform a zero-residual predictor, its predicted residual directions were not reliably above chance, and bounded fusion did not improve held-out questionnaire prediction. The complementary morphology-feature analyses reported in the Supplementary material likewise did not provide a stable incremental pattern. Thus, the present data provide no evidence that the static line drawings add stable incremental predictive information beyond the reduced questionnaire for the assessed 16PF reference scores. Computational feature extraction does not by itself establish the psychological validity of drawing-based indicators (Lilienfeld et al., 2000; Lin et al., 2022).

### 4.4 Implications for dual-process-inspired multimodal modeling

The questionnaire-primary residual architecture was motivated by a dual-process-inspired asymmetry: a direct self-report modality was specified as the primary source of information, while a visual modality was allowed only a bounded residual correction. This is an architectural prior that can be tested, not a validation of dual-process theory and not evidence that the two branches instantiate distinct psychological processes (Evans and Stanovich, 2013). The negative result therefore neither validates nor disproves the broader theoretical framework. It shows only that, in this implementation and sample, the proposed visual candidate modality did not contribute useful residual information.

### 4.5 Why static final drawings may be an insufficient auxiliary modality

A static final drawing preserves some spatial and morphological properties, but it omits the process-level signals that might be informative in other behavioral modalities: stroke timing, pauses, pressure, order, and trajectory dynamics. It also depends on a single end-state image whose content may reflect task interpretation, artistic familiarity, scanning conditions, and paper or lighting variation. Preprocessing can reduce acquisition artifacts; it cannot recover behavioral information that was never recorded. Static drawings should therefore be treated as a deliberately simple exploratory candidate modality rather than an optimal or validated implicit measure (Moeller et al., 2021; Hasan et al., 2023).

### 4.6 Methodological implications for small-sample computational psychology

The study also illustrates why small-sample multimodal prediction requires conservative evaluation. The participant-level outer splits, fold-local response selection, cross-fitted residual construction, and nested scale selection were intended to prevent information leakage and to distinguish apparent visual fit from generalizable incremental value. In clustered, multi-output data, participant-by-factor cells are not independent observations; accordingly, primary comparisons were conducted at the participant level. These safeguards reduce the risk of optimistic estimates, but they cannot compensate for weak or non-generalizable incremental information under the present design.

### 4.7 Limitations

This study has substantial limitations. The sample was small (*N* = 100), and the analysis was exploratory rather than preregistered. The target was a retained Chinese-language 187-item 16PF scoring procedure whose exact adaptation, edition, normative provenance, and licensing status could not be independently verified from the study records. The teacher analysis involved only 15 participants, one familiar rater per participant, and no basis for estimating inter-rater reliability or separating teacher and class effects. The drawing task was also limited to static final images, with heterogeneous acquisition conditions and without process-level recordings. The sample was drawn from high-school and undergraduate educational settings, and detailed participant-level age and sex demographics were not retained. Some paper questionnaires were manually digitized, but detailed transcription quality-control records were not retained. Engagement with the lengthy questionnaire could not be fully verified beyond the original data-cleaning procedures. Teacher nominations did not encode high/low polarity, so that analysis was limited to dimension-level salience.

In addition, the questionnaire subset was data-adaptive and cannot be generalized as a validated short form. Factor-level results are secondary because the 16 outcomes are dependent within participant. The image analyses were confined to the preprocessing and model families evaluated in this study, so the negative result does not demonstrate that every possible visual representation would fail. The residual target is a questionnaire prediction residual, not a latent psychological construct. No independent external dataset was available. The full-form 16PF score is a self-report reference measure, not an objective personality ground truth. Finally, the results concern prediction of the retained full-form reference scores rather than diagnosis, selection, educational decision-making, or any other applied personality assessment use.

### 4.8 Future directions

Future work should begin with larger, independent, and prospectively collected samples; verified instrument provenance; preregistered analytic decisions; and multiple external criteria. Any proposed questionnaire reduction should undergo conventional psychometric validation before being treated as a short form. If drawings remain of interest, researchers should compare static images with prospectively recorded dynamic features and with other low-burden behavioral modalities under standardized acquisition conditions. Such studies should evaluate incremental validity beyond direct measures with conservative participant-level validation and report negative as well as positive results.

## 5 Conclusion

In this exploratory sample, a reduced questionnaire provided a useful but imperfect approximation of retained full-form 16PF reference scores. Static line drawings did not add stable held-out predictive value beyond that questionnaire baseline, and bounded fusion did not improve performance.

These findings do not support using the present static line-drawing modality for personality assessment or decision-making. They instead identify clear requirements for future work: verified measurement provenance, independent psychometric validation, standardized and preferably dynamic behavioral recording, and adequately powered tests of incremental validity.

## Data Availability

The analysis code, configurations, fold definitions, selected-item information, non-sensitive derived outputs, and synthetic pipeline tests supporting this study are publicly available at: https://github.com/qepohaxucesu10-gif/questionnaire-primary-residual-personality and are archived in Zenodo: DOI: 10.5281/zenodo.22748490. Participant-level questionnaire responses, static line drawings, teacher-provided observer records, and private identifier crosswalks are not publicly released because of privacy, consent, and ethical restrictions. Requests for access to restricted participant-level materials can only be considered subject to the applicable ethical approval, consent conditions, and institutional requirements.

## Footnotes

1
https://github.com/qepohaxucesu10-gif/questionnaire-primary-residual-personality

## References

[ref1] Baltrušaitis T. Ahuja C. Morency L.-P. (2019). Multimodal machine learning: a survey and taxonomy. IEEE Trans. Pattern Anal. Mach. Intell. 41, 423–443. doi: 10.1109/TPAMI.2018.2798607 29994351

[ref2] Cattell R. B. Eber H. W. Tatsuoka M. M. (1970). Handbook for the Sixteen Personality Factor Questionnaire (16 PF): In Clinical, Educational, Industrial, and Research Psychology, for Use with All Forms of the Test. Champaign, Illinois: Institute for Personality and Ability Testing.

[ref3] Evans J. S. B. T. Stanovich K. E. (2013). Dual-process theories of higher cognition: advancing the debate. Perspect. Psychol. Sci. 8, 223–241. doi: 10.1177/1745691612460685 26172965

[ref4] Funder D. C. (1995). On the accuracy of personality judgment: a realistic approach. Psychol. Rev. 102, 652–670. doi: 10.1037/0033-295X.102.4.652, 7480467

[ref5] Hasan N. N. Petrides K. V. Hull L. (2023). The relationship between explicit and implicit personality: evidence from the big five and trait emotional intelligence. PLoS One 18:e0287013. doi: 10.1371/journal.pone.0287013, 37812628 PMC10561833

[ref6] Lilienfeld S. O. Wood J. M. Garb H. N. (2000). The scientific status of projective techniques. Psychol. Sci. Public Interest 1, 27–66. doi: 10.1111/1529-1006.002, 26151980

[ref7] Lin Y. Zhang N. Qu Y. Li T. Liu J. Song Y. (2022). The house-tree-person test is not valid for the prediction of mental health: an empirical study using deep neural networks. Acta Psychol. 230:103734. doi: 10.1016/j.actpsy.2022.103734, 36058187

[ref8] Moeller A. N. Johnson B. N. Levy K. N. LeBreton J. M. (2021). “Conceptualizing and measuring the implicit personality: the state of the science,” in Measuring and Modeling Persons and Situations, (London, United Kingdom: Elsevier), 389–426.

[ref9] Paulhus D. L. (1991). “Measurement and control of response bias,” in Measures of Personality and Social Psychological Attitudes, eds. Robinson J. P. Shaver P. R. Wrightsman L. S. (San Diego, California, USA: Academic Press), 17–59.

[ref10] Smith G. T. McCarthy D. M. Anderson K. G. (2000). On the sins of short-form development. Psychol. Assess. 12, 102–111. doi: 10.1037/1040-3590.12.1.102, 10752369

[ref11] Zhao X. Tang Z. Zhang S. (2022). Deep personality trait recognition: a survey. Front. Psychol. 13:839619. doi: 10.3389/fpsyg.2022.839619, 35645923 PMC9136483

