# Supplementary material for: Testing a dual-process-inspired questionnaire-primary residual framework for personality prediction: an exploratory study with static line drawings
Source: Front Psychol. 2026 Oct 8;17:1873771. doi: 10.3389/fpsyg.2026.1873771 (PMC13687008; doi:10.3389/fpsyg.2026.1873771)
Supplement: Supplementary file 1 [file Supplementary_file_1.DOCX]

Supplementary Material

This supplement reports additional descriptive and sensitivity analyses supporting the main analyses. It contains no participant-level raw questionnaire responses, drawings, or participant-teacher linkage records.

# Supplementary Methods

Image preprocessing, fold-local questionnaire selection, cross-fitted residual construction, nested scale selection, and the teacher-provided external comparison followed the analysis protocol described in the main Methods. The primary questionnaire and TinyCNN results below use the same held-out predictions used in the primary analysis. The morphology control is identified separately because it was fitted in a distinct sensitivity analysis.

## **Static morphology features used in the sensitivity analysis**

The separately refitted morphology Ridge sensitivity analysis used label-blind image-derived features. after_foreground_ratio was the fraction of 128 x 128 pixels classified as foreground after preprocessing. after_connected_components was the number of 8-connected foreground components containing at least 2 pixels. after_skeleton_length was the number of foreground pixels in the thinned binary skeleton. after_line_bbox_coverage was the area of the smallest axis-aligned foreground bounding box divided by the 128 x 128 image area. after_edge_pixels was the mean absolute vertical grayscale difference plus the mean absolute horizontal grayscale difference. before_foreground_ratio was the corresponding foreground fraction calculated on the standardized raw grayscale image before clean-gray correction, and before_connected_components was the corresponding 8-connected component count before clean-gray correction. No temporal pause, stroke timing, stroke order, or pen-pressure variable was inferred from the static images. The historical n=40 fragmentation/density analysis is not used as confirmatory evidence in the revised paper.

Table S1. Fold-local questionnaire item selection.

| **Repeat** | **Outer fold** | **Number of selected items** | **Selected questionnaire item IDs** |
| --- | --- | --- | --- |
| 1 | 1 | 32 | Q11;Q13;Q15;Q21;Q27;Q40;Q50;Q51;Q62;Q67;Q72;Q81;Q86;Q93;Q98;Q99;Q105;Q109;Q110;Q127;Q129;Q136;Q139;Q144;Q146;Q156;Q158;Q159;Q167;Q169;Q173;Q183 |
| 1 | 2 | 32 | Q11;Q13;Q14;Q21;Q26;Q32;Q39;Q50;Q51;Q56;Q62;Q67;Q72;Q93;Q102;Q105;Q110;Q114;Q118;Q129;Q132;Q136;Q146;Q147;Q158;Q159;Q167;Q169;Q173;Q174;Q184;Q187 |
| 1 | 3 | 32 | Q11;Q21;Q27;Q32;Q40;Q42;Q51;Q62;Q63;Q67;Q93;Q95;Q98;Q104;Q105;Q109;Q110;Q118;Q127;Q135;Q139;Q140;Q146;Q149;Q156;Q158;Q159;Q171;Q173;Q174;Q182;Q187 |
| 1 | 4 | 32 | Q11;Q14;Q21;Q27;Q30;Q32;Q37;Q38;Q40;Q50;Q51;Q67;Q72;Q75;Q83;Q86;Q89;Q93;Q94;Q105;Q109;Q110;Q123;Q127;Q135;Q146;Q156;Q157;Q159;Q167;Q169;Q173 |
| 1 | 5 | 32 | Q11;Q17;Q21;Q32;Q38;Q39;Q40;Q50;Q51;Q62;Q63;Q67;Q76;Q79;Q81;Q93;Q102;Q109;Q110;Q118;Q129;Q134;Q135;Q146;Q147;Q158;Q169;Q171;Q173;Q174;Q183;Q187 |
| 2 | 1 | 32 | Q21;Q23;Q26;Q27;Q32;Q39;Q40;Q50;Q51;Q62;Q63;Q67;Q72;Q93;Q95;Q105;Q109;Q110;Q112;Q118;Q129;Q135;Q139;Q142;Q146;Q149;Q156;Q157;Q159;Q173;Q182;Q187 |
| 2 | 2 | 32 | Q12;Q27;Q30;Q32;Q38;Q39;Q40;Q51;Q62;Q63;Q67;Q72;Q81;Q93;Q95;Q99;Q109;Q110;Q118;Q127;Q129;Q132;Q134;Q135;Q146;Q147;Q167;Q169;Q173;Q174;Q182;Q187 |
| 2 | 3 | 32 | Q11;Q13;Q14;Q21;Q27;Q32;Q37;Q40;Q41;Q50;Q51;Q67;Q83;Q93;Q105;Q109;Q110;Q118;Q123;Q127;Q129;Q136;Q139;Q146;Q156;Q159;Q169;Q171;Q173;Q174;Q182;Q187 |
| 2 | 4 | 32 | Q11;Q13;Q17;Q21;Q32;Q40;Q51;Q59;Q62;Q63;Q72;Q74;Q77;Q81;Q83;Q93;Q94;Q105;Q109;Q110;Q115;Q127;Q129;Q135;Q146;Q147;Q151;Q167;Q169;Q173;Q174;Q183 |
| 2 | 5 | 32 | Q11;Q15;Q17;Q21;Q27;Q32;Q40;Q48;Q50;Q51;Q62;Q63;Q72;Q93;Q95;Q102;Q105;Q109;Q110;Q114;Q117;Q127;Q129;Q136;Q146;Q156;Q158;Q159;Q168;Q173;Q174;Q183 |
| 3 | 1 | 32 | Q15;Q21;Q27;Q32;Q37;Q40;Q50;Q56;Q62;Q63;Q75;Q76;Q83;Q86;Q93;Q98;Q105;Q109;Q110;Q117;Q129;Q134;Q135;Q139;Q143;Q146;Q158;Q167;Q169;Q171;Q173;Q187 |
| 3 | 2 | 32 | Q21;Q27;Q30;Q32;Q39;Q40;Q50;Q51;Q62;Q64;Q67;Q69;Q72;Q93;Q105;Q109;Q110;Q127;Q135;Q139;Q142;Q146;Q147;Q149;Q156;Q159;Q162;Q169;Q173;Q182;Q183;Q187 |
| 3 | 3 | 32 | Q11;Q12;Q16;Q27;Q32;Q38;Q40;Q42;Q50;Q51;Q60;Q63;Q70;Q72;Q73;Q81;Q93;Q95;Q102;Q105;Q109;Q110;Q127;Q129;Q146;Q159;Q166;Q168;Q173;Q174;Q182;Q183 |
| 3 | 4 | 32 | Q11;Q14;Q17;Q21;Q23;Q27;Q32;Q38;Q40;Q50;Q51;Q62;Q63;Q67;Q72;Q83;Q86;Q93;Q95;Q105;Q109;Q110;Q127;Q129;Q135;Q144;Q146;Q156;Q159;Q173;Q174;Q183 |
| 3 | 5 | 32 | Q21;Q26;Q27;Q32;Q37;Q40;Q50;Q51;Q62;Q63;Q67;Q72;Q93;Q98;Q102;Q105;Q109;Q110;Q114;Q118;Q129;Q132;Q135;Q141;Q142;Q146;Q156;Q157;Q159;Q169;Q173;Q174 |
| 4 | 1 | 32 | Q12;Q21;Q26;Q33;Q38;Q40;Q50;Q51;Q62;Q63;Q67;Q72;Q81;Q93;Q95;Q98;Q102;Q105;Q109;Q110;Q127;Q129;Q135;Q142;Q144;Q146;Q156;Q159;Q166;Q173;Q174;Q183 |
| 4 | 2 | 32 | Q14;Q17;Q26;Q27;Q37;Q39;Q50;Q51;Q62;Q63;Q67;Q68;Q72;Q79;Q81;Q93;Q95;Q98;Q105;Q109;Q110;Q132;Q135;Q139;Q146;Q156;Q159;Q169;Q173;Q174;Q182;Q187 |
| 4 | 3 | 32 | Q11;Q13;Q14;Q16;Q21;Q27;Q32;Q39;Q50;Q51;Q63;Q67;Q72;Q83;Q93;Q99;Q105;Q109;Q110;Q123;Q127;Q129;Q135;Q137;Q146;Q156;Q160;Q168;Q169;Q173;Q182;Q187 |
| 4 | 4 | 31 | Q11;Q15;Q17;Q26;Q27;Q32;Q40;Q50;Q62;Q67;Q72;Q86;Q89;Q93;Q98;Q105;Q109;Q114;Q118;Q120;Q129;Q135;Q146;Q155;Q158;Q159;Q169;Q173;Q174;Q183;Q187 |
| 4 | 5 | 32 | Q11;Q21;Q27;Q32;Q37;Q40;Q48;Q51;Q63;Q67;Q72;Q75;Q93;Q104;Q105;Q109;Q110;Q118;Q127;Q136;Q139;Q140;Q146;Q155;Q158;Q159;Q167;Q169;Q173;Q174;Q182;Q187 |
| 5 | 1 | 32 | Q11;Q15;Q21;Q27;Q30;Q32;Q40;Q42;Q50;Q51;Q62;Q64;Q67;Q72;Q73;Q86;Q93;Q99;Q105;Q109;Q110;Q114;Q127;Q133;Q146;Q156;Q159;Q168;Q169;Q173;Q183;Q187 |
| 5 | 2 | 32 | Q11;Q13;Q14;Q16;Q21;Q26;Q27;Q32;Q37;Q40;Q67;Q86;Q93;Q95;Q98;Q99;Q105;Q109;Q110;Q118;Q129;Q135;Q139;Q146;Q156;Q158;Q159;Q171;Q173;Q174;Q182;Q187 |
| 5 | 3 | 32 | Q11;Q21;Q27;Q32;Q39;Q40;Q50;Q51;Q62;Q64;Q67;Q68;Q72;Q81;Q83;Q86;Q98;Q105;Q109;Q110;Q117;Q118;Q129;Q135;Q139;Q145;Q146;Q159;Q173;Q174;Q178;Q183 |
| 5 | 4 | 32 | Q11;Q15;Q27;Q32;Q40;Q48;Q50;Q56;Q62;Q63;Q67;Q72;Q76;Q93;Q95;Q105;Q109;Q110;Q114;Q118;Q127;Q129;Q136;Q146;Q167;Q169;Q173;Q174;Q182;Q183;Q184;Q187 |
| 5 | 5 | 32 | Q11;Q14;Q17;Q21;Q22;Q38;Q40;Q51;Q63;Q81;Q93;Q105;Q109;Q110;Q117;Q127;Q129;Q136;Q137;Q144;Q146;Q147;Q149;Q156;Q157;Q158;Q159;Q169;Q173;Q174;Q176;Q187 |

Table S2. Questionnaire item-selection frequency.

| **Questionnaire item** | **Number of outer folds selected** | **Proportion of 25 outer folds** | **Formal 16PF factor-scoring item?** |
| --- | --- | --- | --- |
| Q11 | 18 | 0.720 | Yes — retained formal factor-scoring item |
| Q12 | 3 | 0.120 | Yes — retained formal factor-scoring item |
| Q13 | 6 | 0.240 | Yes — retained formal factor-scoring item |
| Q14 | 8 | 0.320 | Yes — retained formal factor-scoring item |
| Q15 | 6 | 0.240 | Yes — retained formal factor-scoring item |
| Q16 | 3 | 0.120 | Yes — retained formal factor-scoring item |
| Q17 | 7 | 0.280 | Yes — retained formal factor-scoring item |
| Q21 | 20 | 0.800 | Yes — retained formal factor-scoring item |
| Q22 | 1 | 0.040 | Yes — retained formal factor-scoring item |
| Q23 | 2 | 0.080 | Yes — retained formal factor-scoring item |
| Q26 | 7 | 0.280 | Yes — retained formal factor-scoring item |
| Q27 | 20 | 0.800 | Yes — retained formal factor-scoring item |
| Q30 | 4 | 0.160 | Yes — retained formal factor-scoring item |
| Q32 | 21 | 0.840 | Yes — retained formal factor-scoring item |
| Q33 | 1 | 0.040 | Yes — retained formal factor-scoring item |
| Q37 | 7 | 0.280 | Yes — retained formal factor-scoring item |
| Q38 | 7 | 0.280 | Yes — retained formal factor-scoring item |
| Q39 | 8 | 0.320 | Yes — retained formal factor-scoring item |
| Q40 | 22 | 0.880 | Yes — retained formal factor-scoring item |
| Q41 | 1 | 0.040 | Yes — retained formal factor-scoring item |
| Q42 | 3 | 0.120 | Yes — retained formal factor-scoring item |
| Q48 | 3 | 0.120 | Yes — retained formal factor-scoring item |
| Q50 | 19 | 0.760 | Yes — retained formal factor-scoring item |
| Q51 | 21 | 0.840 | Yes — retained formal factor-scoring item |
| Q56 | 3 | 0.120 | Yes — retained formal factor-scoring item |
| Q59 | 1 | 0.040 | Yes — retained formal factor-scoring item |
| Q60 | 1 | 0.040 | Yes — retained formal factor-scoring item |
| Q62 | 18 | 0.720 | Yes — retained formal factor-scoring item |
| Q63 | 16 | 0.640 | Yes — retained formal factor-scoring item |
| Q64 | 3 | 0.120 | Yes — retained formal factor-scoring item |
| Q67 | 20 | 0.800 | Yes — retained formal factor-scoring item |
| Q68 | 2 | 0.080 | Yes — retained formal factor-scoring item |
| Q69 | 1 | 0.040 | Yes — retained formal factor-scoring item |
| Q70 | 1 | 0.040 | Yes — retained formal factor-scoring item |
| Q72 | 19 | 0.760 | Yes — retained formal factor-scoring item |
| Q73 | 2 | 0.080 | Yes — retained formal factor-scoring item |
| Q74 | 1 | 0.040 | Yes — retained formal factor-scoring item |
| Q75 | 3 | 0.120 | Yes — retained formal factor-scoring item |
| Q76 | 3 | 0.120 | Yes — retained formal factor-scoring item |
| Q77 | 1 | 0.040 | Yes — retained formal factor-scoring item |
| Q79 | 2 | 0.080 | Yes — retained formal factor-scoring item |
| Q81 | 9 | 0.360 | Yes — retained formal factor-scoring item |
| Q83 | 7 | 0.280 | Yes — retained formal factor-scoring item |
| Q86 | 8 | 0.320 | Yes — retained formal factor-scoring item |
| Q89 | 2 | 0.080 | Yes — retained formal factor-scoring item |
| Q93 | 24 | 0.960 | Yes — retained formal factor-scoring item |
| Q94 | 2 | 0.080 | Yes — retained formal factor-scoring item |
| Q95 | 10 | 0.400 | Yes — retained formal factor-scoring item |
| Q98 | 9 | 0.360 | Yes — retained formal factor-scoring item |
| Q99 | 5 | 0.200 | Yes — retained formal factor-scoring item |
| Q102 | 6 | 0.240 | Yes — retained formal factor-scoring item |
| Q104 | 2 | 0.080 | Yes — retained formal factor-scoring item |
| Q105 | 23 | 0.920 | Yes — retained formal factor-scoring item |
| Q109 | 24 | 0.960 | Yes — retained formal factor-scoring item |
| Q110 | 24 | 0.960 | Yes — retained formal factor-scoring item |
| Q112 | 1 | 0.040 | Yes — retained formal factor-scoring item |
| Q114 | 6 | 0.240 | Yes — retained formal factor-scoring item |
| Q115 | 1 | 0.040 | Yes — retained formal factor-scoring item |
| Q117 | 4 | 0.160 | Yes — retained formal factor-scoring item |
| Q118 | 12 | 0.480 | Yes — retained formal factor-scoring item |
| Q120 | 1 | 0.040 | Yes — retained formal factor-scoring item |
| Q123 | 3 | 0.120 | Yes — retained formal factor-scoring item |
| Q127 | 16 | 0.640 | Yes — retained formal factor-scoring item |
| Q129 | 19 | 0.760 | Yes — retained formal factor-scoring item |
| Q132 | 4 | 0.160 | Yes — retained formal factor-scoring item |
| Q133 | 1 | 0.040 | Yes — retained formal factor-scoring item |
| Q134 | 3 | 0.120 | Yes — retained formal factor-scoring item |
| Q135 | 16 | 0.640 | Yes — retained formal factor-scoring item |
| Q136 | 7 | 0.280 | Yes — retained formal factor-scoring item |
| Q137 | 2 | 0.080 | Yes — retained formal factor-scoring item |
| Q139 | 10 | 0.400 | Yes — retained formal factor-scoring item |
| Q140 | 2 | 0.080 | Yes — retained formal factor-scoring item |
| Q141 | 1 | 0.040 | Yes — retained formal factor-scoring item |
| Q142 | 4 | 0.160 | Yes — retained formal factor-scoring item |
| Q143 | 1 | 0.040 | Yes — retained formal factor-scoring item |
| Q144 | 4 | 0.160 | Yes — retained formal factor-scoring item |
| Q145 | 1 | 0.040 | Yes — retained formal factor-scoring item |
| Q146 | 25 | 1.000 | Yes — retained formal factor-scoring item |
| Q147 | 6 | 0.240 | Yes — retained formal factor-scoring item |
| Q149 | 4 | 0.160 | Yes — retained formal factor-scoring item |
| Q151 | 1 | 0.040 | Yes — retained formal factor-scoring item |
| Q155 | 2 | 0.080 | Yes — retained formal factor-scoring item |
| Q156 | 15 | 0.600 | Yes — retained formal factor-scoring item |
| Q157 | 4 | 0.160 | Yes — retained formal factor-scoring item |
| Q158 | 10 | 0.400 | Yes — retained formal factor-scoring item |
| Q159 | 19 | 0.760 | Yes — retained formal factor-scoring item |
| Q160 | 1 | 0.040 | Yes — retained formal factor-scoring item |
| Q162 | 1 | 0.040 | Yes — retained formal factor-scoring item |
| Q166 | 2 | 0.080 | Yes — retained formal factor-scoring item |
| Q167 | 8 | 0.320 | Yes — retained formal factor-scoring item |
| Q168 | 4 | 0.160 | Yes — retained formal factor-scoring item |
| Q169 | 17 | 0.680 | Yes — retained formal factor-scoring item |
| Q171 | 5 | 0.200 | Yes — retained formal factor-scoring item |
| Q173 | 25 | 1.000 | Yes — retained formal factor-scoring item |
| Q174 | 18 | 0.720 | Yes — retained formal factor-scoring item |
| Q176 | 1 | 0.040 | Yes — retained formal factor-scoring item |
| Q178 | 1 | 0.040 | Yes — retained formal factor-scoring item |
| Q182 | 11 | 0.440 | Yes — retained formal factor-scoring item |
| Q183 | 12 | 0.480 | Yes — retained formal factor-scoring item |
| Q184 | 2 | 0.080 | Yes — retained formal factor-scoring item |
| Q187 | 16 | 0.640 | No — not a formal factor-scoring item |

Table S3. Pairwise selected-item-set stability.

| **Number of folds** | **Mean pairwise Jaccard** | **SD pairwise Jaccard** | **Minimum pairwise Jaccard** | **Maximum pairwise Jaccard** | **Minimum items per fold** | **Maximum items per fold** | **Questionnaire item 1 selection frequency** | **Questionnaire item 2 selection frequency** | **Questionnaire item 187 selection frequency** |
| --- | --- | --- | --- | --- | --- | --- | --- | --- | --- |
| 25 | 0.414 | 0.068 | 0.231 | 0.600 | 31 | 32 | 0 | 0 | 16 |

Table S4. Factor-level questionnaire correspondence from the final primary pipeline.

| **Factor** | **Participants** | **Pearson r** | **Pearson r 95% CI lower** | **Pearson r 95% CI upper** | **Spearman rho** | **MAE** | **RMSE** | **R²** |
| --- | --- | --- | --- | --- | --- | --- | --- | --- |
| A | 100 | 0.647 | 0.509 | 0.757 | 0.645 | 1.006 | 1.280 | 0.400 |
| B | 100 | 0.341 | 0.130 | 0.524 | 0.282 | 1.384 | 1.702 | 0.114 |
| C | 100 | 0.653 | 0.526 | 0.756 | 0.631 | 0.939 | 1.168 | 0.420 |
| E | 100 | 0.660 | 0.521 | 0.770 | 0.648 | 0.929 | 1.261 | 0.426 |
| F | 100 | 0.677 | 0.568 | 0.765 | 0.640 | 1.136 | 1.415 | 0.434 |
| G | 100 | 0.627 | 0.486 | 0.743 | 0.619 | 1.075 | 1.365 | 0.364 |
| H | 100 | 0.775 | 0.682 | 0.847 | 0.756 | 1.041 | 1.257 | 0.583 |
| I | 100 | 0.587 | 0.444 | 0.703 | 0.553 | 1.235 | 1.541 | 0.331 |
| L | 100 | 0.606 | 0.473 | 0.715 | 0.545 | 1.174 | 1.398 | 0.352 |
| M | 100 | 0.567 | 0.421 | 0.682 | 0.533 | 0.994 | 1.266 | 0.312 |
| N | 100 | 0.384 | 0.215 | 0.542 | 0.368 | 1.183 | 1.427 | 0.145 |
| O | 100 | 0.645 | 0.516 | 0.749 | 0.629 | 1.307 | 1.554 | 0.403 |
| Q1 | 100 | 0.510 | 0.383 | 0.625 | 0.519 | 1.333 | 1.612 | 0.255 |
| Q2 | 100 | 0.716 | 0.615 | 0.804 | 0.729 | 1.180 | 1.468 | 0.488 |
| Q3 | 100 | 0.485 | 0.322 | 0.632 | 0.461 | 0.957 | 1.228 | 0.233 |
| Q4 | 100 | 0.686 | 0.580 | 0.774 | 0.702 | 0.919 | 1.172 | 0.455 |

Table S5a. Factor-level visual residual results from the final primary pipeline.

| **Factor** | **Predictor** | **Observations** | **Residual MAE** | **Residual MSE** | **Direction accuracy (\|residual\| ≥ .25)** | **Balanced accuracy (\|residual\| ≥ .25)** | **Pearson r** | **Spearman rho** |
| --- | --- | --- | --- | --- | --- | --- | --- | --- |
| Overall | Zero residual | 1600 | 1.112 | 1.935 | — | — | — | — |
| Overall | TinyCNN | 1600 | 1.227 | 2.364 | 0.486 | 0.486 | -0.055 | -0.048 |
| A | Zero residual | 100 | 1.006 | 1.639 | — | — | — | — |
| A | TinyCNN | 100 | 1.144 | 2.045 | 0.390 | 0.401 | -0.147 | -0.226 |
| B | Zero residual | 100 | 1.384 | 2.896 | — | — | — | — |
| B | TinyCNN | 100 | 1.523 | 3.430 | 0.484 | 0.485 | -0.171 | -0.163 |
| C | Zero residual | 100 | 0.939 | 1.364 | — | — | — | — |
| C | TinyCNN | 100 | 1.040 | 1.576 | 0.540 | 0.547 | 0.095 | 0.091 |
| E | Zero residual | 100 | 0.929 | 1.590 | — | — | — | — |
| E | TinyCNN | 100 | 1.067 | 2.022 | 0.547 | 0.555 | -0.012 | 0.049 |
| F | Zero residual | 100 | 1.136 | 2.001 | — | — | — | — |
| F | TinyCNN | 100 | 1.248 | 2.351 | 0.483 | 0.480 | -0.030 | -0.011 |
| G | Zero residual | 100 | 1.075 | 1.862 | — | — | — | — |
| G | TinyCNN | 100 | 1.210 | 2.476 | 0.547 | 0.540 | 0.049 | 0.078 |
| H | Zero residual | 100 | 1.041 | 1.580 | — | — | — | — |
| H | TinyCNN | 100 | 1.131 | 1.890 | 0.471 | 0.473 | -0.073 | -0.084 |
| I | Zero residual | 100 | 1.235 | 2.376 | — | — | — | — |
| I | TinyCNN | 100 | 1.439 | 3.028 | 0.470 | 0.467 | -0.035 | -0.035 |
| L | Zero residual | 100 | 1.174 | 1.954 | — | — | — | — |
| L | TinyCNN | 100 | 1.263 | 2.388 | 0.489 | 0.488 | -0.138 | -0.156 |
| M | Zero residual | 100 | 0.994 | 1.603 | — | — | — | — |
| M | TinyCNN | 100 | 1.066 | 1.827 | 0.416 | 0.441 | -0.072 | -0.074 |
| N | Zero residual | 100 | 1.183 | 2.036 | — | — | — | — |
| N | TinyCNN | 100 | 1.311 | 2.574 | 0.471 | 0.473 | -0.092 | -0.076 |
| O | Zero residual | 100 | 1.307 | 2.415 | — | — | — | — |
| O | TinyCNN | 100 | 1.354 | 2.822 | 0.533 | 0.529 | 0.017 | 0.025 |
| Q1 | Zero residual | 100 | 1.333 | 2.598 | — | — | — | — |
| Q1 | TinyCNN | 100 | 1.535 | 3.275 | 0.407 | 0.418 | -0.192 | -0.160 |
| Q2 | Zero residual | 100 | 1.180 | 2.155 | — | — | — | — |
| Q2 | TinyCNN | 100 | 1.241 | 2.481 | 0.539 | 0.539 | -0.000 | 0.048 |
| Q3 | Zero residual | 100 | 0.957 | 1.509 | — | — | — | — |
| Q3 | TinyCNN | 100 | 1.109 | 2.089 | 0.464 | 0.466 | -0.198 | -0.149 |
| Q4 | Zero residual | 100 | 0.919 | 1.373 | — | — | — | — |
| Q4 | TinyCNN | 100 | 0.959 | 1.556 | 0.537 | 0.539 | 0.033 | 0.059 |

This morphology control was fitted in a separate sensitivity analysis using cross-fitted residual targets. Its numerical baseline is therefore not treated as the same stochastic realization as the primary analysis.

Table S5b. Separately refitted morphology-based sensitivity analysis.

| **Analysis scope** | **Predictor** | **Observations** | **Residual MAE** | **Residual MSE** | **Direction accuracy (\|residual\| ≥ .25)** | **Balanced accuracy (\|residual\| ≥ .25)** | **Pearson r** | **Spearman rho** |
| --- | --- | --- | --- | --- | --- | --- | --- | --- |
| overall | Zero residual | 1600 | 1.105 | 1.917 | — | — | — | — |
| overall | TinyCNN | 1600 | 1.222 | 2.376 | 0.487 | 0.487 | -0.050 | -0.033 |
| overall | Morphology Ridge | 1600 | 1.141 | 2.061 | 0.489 | 0.489 | -0.037 | -0.035 |
| A | Zero residual | 100 | 0.978 | 1.556 | — | — | — | — |
| A | TinyCNN | 100 | 1.134 | 2.032 | 0.427 | 0.431 | -0.283 | -0.257 |
| A | Morphology Ridge | 100 | 1.030 | 1.676 | 0.415 | 0.431 | -0.055 | -0.114 |
| B | Zero residual | 100 | 1.323 | 2.661 | — | — | — | — |
| B | TinyCNN | 100 | 1.487 | 3.383 | 0.457 | 0.446 | -0.201 | -0.188 |
| B | Morphology Ridge | 100 | 1.356 | 3.055 | 0.554 | 0.543 | -0.140 | -0.057 |
| C | Zero residual | 100 | 0.951 | 1.452 | — | — | — | — |
| C | TinyCNN | 100 | 1.026 | 1.808 | 0.529 | 0.546 | 0.037 | 0.094 |
| C | Morphology Ridge | 100 | 0.961 | 1.551 | 0.586 | 0.584 | 0.017 | 0.040 |
| E | Zero residual | 100 | 0.937 | 1.503 | — | — | — | — |
| E | TinyCNN | 100 | 1.145 | 2.100 | 0.388 | 0.397 | -0.110 | -0.078 |
| E | Morphology Ridge | 100 | 1.012 | 1.705 | 0.459 | 0.461 | -0.202 | -0.212 |
| F | Zero residual | 100 | 1.122 | 1.968 | — | — | — | — |
| F | TinyCNN | 100 | 1.266 | 2.470 | 0.422 | 0.418 | -0.166 | -0.167 |
| F | Morphology Ridge | 100 | 1.179 | 2.210 | 0.444 | 0.434 | -0.196 | -0.193 |
| G | Zero residual | 100 | 1.102 | 1.924 | — | — | — | — |
| G | TinyCNN | 100 | 1.201 | 2.520 | 0.562 | 0.560 | -0.005 | 0.077 |
| G | Morphology Ridge | 100 | 1.160 | 2.125 | 0.427 | 0.427 | -0.252 | -0.268 |
| H | Zero residual | 100 | 1.026 | 1.507 | — | — | — | — |
| H | TinyCNN | 100 | 1.023 | 1.639 | 0.612 | 0.615 | 0.132 | 0.152 |
| H | Morphology Ridge | 100 | 1.032 | 1.515 | 0.553 | 0.555 | 0.125 | 0.135 |
| I | Zero residual | 100 | 1.262 | 2.457 | — | — | — | — |
| I | TinyCNN | 100 | 1.329 | 2.612 | 0.614 | 0.612 | 0.218 | 0.291 |
| I | Morphology Ridge | 100 | 1.300 | 2.626 | 0.506 | 0.504 | -0.006 | -0.031 |
| L | Zero residual | 100 | 1.218 | 2.104 | — | — | — | — |
| L | TinyCNN | 100 | 1.338 | 2.671 | 0.440 | 0.442 | -0.294 | -0.263 |
| L | Morphology Ridge | 100 | 1.251 | 2.252 | 0.451 | 0.452 | -0.016 | -0.042 |
| M | Zero residual | 100 | 0.975 | 1.581 | — | — | — | — |
| M | TinyCNN | 100 | 1.096 | 1.903 | 0.405 | 0.403 | -0.159 | -0.196 |
| M | Morphology Ridge | 100 | 1.018 | 1.692 | 0.440 | 0.449 | -0.090 | -0.114 |
| N | Zero residual | 100 | 1.134 | 1.875 | — | — | — | — |
| N | TinyCNN | 100 | 1.253 | 2.585 | 0.523 | 0.536 | 0.012 | 0.004 |
| N | Morphology Ridge | 100 | 1.190 | 2.054 | 0.442 | 0.438 | -0.224 | -0.177 |
| O | Zero residual | 100 | 1.285 | 2.435 | — | — | — | — |
| O | TinyCNN | 100 | 1.377 | 2.951 | 0.552 | 0.553 | -0.042 | -0.014 |
| O | Morphology Ridge | 100 | 1.336 | 2.670 | 0.471 | 0.471 | -0.083 | -0.078 |
| Q1 | Zero residual | 100 | 1.302 | 2.599 | — | — | — | — |
| Q1 | TinyCNN | 100 | 1.430 | 3.067 | 0.462 | 0.435 | -0.067 | -0.065 |
| Q1 | Morphology Ridge | 100 | 1.240 | 2.601 | 0.571 | 0.564 | 0.130 | 0.165 |
| Q2 | Zero residual | 100 | 1.158 | 2.074 | — | — | — | — |
| Q2 | TinyCNN | 100 | 1.163 | 2.113 | 0.598 | 0.595 | 0.164 | 0.185 |
| Q2 | Morphology Ridge | 100 | 1.184 | 2.066 | 0.524 | 0.521 | 0.160 | 0.137 |
| Q3 | Zero residual | 100 | 0.943 | 1.471 | — | — | — | — |
| Q3 | TinyCNN | 100 | 1.136 | 2.092 | 0.366 | 0.366 | -0.254 | -0.311 |
| Q3 | Morphology Ridge | 100 | 0.975 | 1.570 | 0.512 | 0.512 | -0.040 | -0.026 |
| Q4 | Zero residual | 100 | 0.964 | 1.510 | — | — | — | — |
| Q4 | TinyCNN | 100 | 1.149 | 2.067 | 0.434 | 0.432 | -0.151 | -0.161 |
| Q4 | Morphology Ridge | 100 | 1.028 | 1.614 | 0.458 | 0.459 | -0.053 | -0.072 |

Table S6. Fixed-scale sensitivity and nested-selection frequency.

| **Scale** | **Mean outer-fold MAE** | **SD across outer folds** | **Folds lower MAE than α = 0** | **Nested-selection frequency** |
| --- | --- | --- | --- | --- |
| 0.000 | 1.210 | 0.063 | 0 | 21 |
| 0.005 | 1.210 | 0.063 | 8 | 0 |
| 0.010 | 1.210 | 0.063 | 8 | 1 |
| 0.025 | 1.211 | 0.063 | 8 | 2 |
| 0.050 | 1.212 | 0.063 | 8 | 1 |
| 0.075 | 1.213 | 0.063 | 7 | 0 |
| 0.100 | 1.215 | 0.063 | 6 | 0 |

Table S7. Teacher-provided external-criterion summary.

| **Method** | **Participants** | **Mean overlap** | **Any hit** | **Exact match** | **Jaccard** | **Precision at 3** | **Mean-overlap CI lower** | **Mean-overlap CI upper** |
| --- | --- | --- | --- | --- | --- | --- | --- | --- |
| Full-form self-report reference | 15 | 0.800 | 0.600 | 0.000 | 0.180 | 0.267 | 0.400 | 1.200 |
| Questionnaire-only | 15 | 0.467 | 0.333 | 0.000 | 0.107 | 0.156 | 0.133 | 0.867 |
| Fixed α = .05 fusion | 15 | 0.467 | 0.333 | 0.000 | 0.107 | 0.156 | 0.133 | 0.867 |
| Nested-selected fusion | 15 | 0.467 | 0.333 | 0.000 | 0.107 | 0.156 | 0.133 | 0.867 |

**Table S8. Feature and target flow in the revised framework.**

| **Input/source** | **Representation** | **Fold-local processing** | **Model/criterion** | **Training target** | **Output** | **Role** |
| --- | --- | --- | --- | --- | --- | --- |
| Full-form reference scores | 16 factor Sten scores | Formal scoring uses retained item-specific keys and conversion table | Questionnaire Tower; residual construction | Questionnaire prediction target; combined with OOF questionnaire predictions to construct the residual target. | Reference score vector | Scoring reference |
| Reduced questionnaire response vector | 31-32 selected A=2, B=1, C=0 features | Selection and standardization within training partition only | Questionnaire Tower | Normalized-score MSE | 16 questionnaire predictions | Primary predictive anchor |
| Static clean-gray drawing | 1 x 128 x 128 standardized grayscale image | Fixed label-blind preprocessing; no outcome labels | TinyCNN | Cross-fitted questionnaire residual; Sten-scale MSE | 16 visual residual predictions | Bounded auxiliary candidate |
| Teacher-provided observer criterion | Three nominated salient dimensions from 16-factor set | No model fitting; mapped cases only | External observer comparison | Not applicable | Overlap, any-hit, exact-match, and Jaccard summaries | Secondary external criterion |

# Supplementary Figures

Figure S2. Cross-fitted residual-target distributions from the separately refitted diagnostic analysis.

Figure S3. Residual MAE from the separately refitted morphology sensitivity analysis. This analysis is distinct from the primary visual-residual analysis.
